# Occipital Stroke Due to Non-bacterial Thrombotic Endocarditis as the Initial Manifestation of Endometrioid Ovarian Cancer

**DOI:** 10.7759/cureus.90521

**Published:** 2025-08-19

**Authors:** Aparna Mohapatra, Ansy Patel, Vivek Bhat, Ganaraja V Harikrishna, Suresha Kodapala

**Affiliations:** 1 General Medicine, St. John’s Medical College, Bangalore, IND; 2 Medicine, State University of New York Upstate Medical University, Syracuse, USA; 3 Neurology, National Institute of Mental Health and Neurosciences, Bangalore, IND; 4 Neurology, Sri Madhusudan Sai Institute of Medical Sciences and Research, Muddenahalli, IND; 5 Neurology, RxDx Healthcare, Bangalore, IND

**Keywords:** hypercoagulable state, ischemic stroke, non-infective endocarditis, occult malignancy, thromboembolism

## Abstract

Ischemic stroke is a rare initial manifestation of malignancy. We report the case of a 46-year-old female who presented with an embolic stroke, subsequently found to be due to endometrioid ovarian carcinoma. She presented to our clinic with sudden-onset bilateral visual blurring and syncope. Neurological examination was unremarkable. Investigations revealed iron-deficiency anemia and elevated C-reactive protein. Brain MRI showed multiple cardioembolic infarcts in the bilateral frontoparietal lobes and cerebellum. transesophageal echocardiogram (TEE) revealed a left atrial mass attached to the posterior mitral leaflet, suggestive of non-bacterial thrombotic endocarditis (NBTE). Further workup, with CT of the abdomen and pelvis, finally revealed a solid-cystic right ovarian mass, suggestive of a neoplasm, with right iliac lymphadenopathy and left ovarian endometriotic cysts, along with elevated cancer antigen (CA)-125 levels. The patient underwent bilateral salpingo-oophorectomy, with surgical staging confirming grade-2 endometrioid adenocarcinoma. She received chemotherapy (paclitaxel/ carboplatin) for stage 3 disease, following which she improved clinically and her cancer antigen (CA)-125 levels normalized.

This report highlights stroke due to NBTE as a rare initial presentation of endometrioid ovarian carcinoma, a histologic subtype not known for hypercoagulability. It emphasizes the need to consider occult malignancy in young patients presenting with embolic stroke in the absence of conventional vascular risk factors. Clues towards occult malignancy included unexplained anemia, elevated inflammatory markers, and the "Three Territory Sign" (TTS) on MRI. Furthermore, the report adds to the literature regarding NBTE, an underrecognized entity strongly associated with cancer. A high degree of suspicion, along with a systematic approach to workup, can facilitate early diagnosis in these patients.

## Introduction

While the association between solid tumors and venous thromboembolism (VTE) is well-known, arterial events are less common. Stroke as the initial manifestation of an occult neoplasm remains rare [1], and detection of the neoplasm is often challenging in the absence of clear systemic signs [2]. We report the case of a middle-aged, previously healthy woman who presented with multiple posterior circulation emboli as the first manifestation of an ovarian malignancy.

## Case presentation

A 46-year-old female, an educated, working professional with no known comorbidities, presented to our Neurology clinic. She reported complaints of sudden-onset blurring of vision in both eyes and one episode of loss of consciousness. She had no incoordination, limb weakness, involuntary movements, or incontinence. System review revealed occasional lower abdominal pain and heavy menstrual bleeding. She denied palpitations, chest pain, or syncope. She also denied other significant past medical history or family history. Neurological and systemic examination, including ophthalmological examination, was unremarkable.

Initial investigations were suggestive of iron deficiency anemia. Additionally, she had an increased total leukocyte count and a highly elevated serum C-reactive protein level. She had normal blood sugar levels, lipid profile, and coagulation profile (Table 1).

**Table 1:** Results of initial investigations ALP: alkaline phosphatase; ALT: alanine aminotransferase; AST: aspartate aminotransferase; CRP: C-reactive protein; GGT: gamma-glutamyl transferase; HbA1c: hemoglobin A1c; HDL: high-density lipoprotein; LDL: low-density lipoprotein; TIBC: total iron-binding capacity; TSH: thyroid-stimulating hormone; VLDL: very low-density lipoprotein

Investigation	Observed value	Reference range
Hemoglobin (g/dL)	7.3	11.5-16
White cell count (/mm^3^)	16,590	4,000-11,000
Neutrophils (%)	86.5	40-75
Lymphocytes (%)	8.8	20-45
Hematocrit (%)	23.6	42-52
Platelet count (%)	2.49	1.5-5
Cholesterol (mg/dL)	194	Normal: <200; borderline: 200-239; high: ≥240
Triglycerides (mg/dL)	146	Normal: <150; borderline: 150-199; high: ≥200
HDL (mg/dL)	33.6	Low: <40; normal: 40-59; high ≥60
LDL (mg/dL)	131.2	Good: <100; near-optimal: 100-129; borderline: 130-159
VLDL (mg/dL)	29.2	10.0-40.0
Total bilirubin (mg/dL)	0.4	0.1-1.4
Direct bilirubin (mg/dL)	0.1	0.0-0.4
Total protein (g/dL)	7.2	6.4-8.3
Albumin (g/dL)	4.1	3.5-5.5
Globulin (g/dL)	3.1	1.8-3.4
AST (U/L)	11	0-45
ALT (U/L)	17	0-45
ALP (U/L)	92	0-115
GGT (U/L)	28	0-50
HbA1c (%)	6.6	Non-diabetic: <6; prediabetic: 6-7
Vitamin B12 (pg/mL)	317.1	210-950
Iron (ug/dL)	19.8	37-145
TIBC (ug/dL)	419.2	228-428
Ferritin (mcg/L)	19.71	13-150
TSH (uIU/mL)	0.33	0.27-5.0
CRP (mg/L)	15.62	1.0-5.0
Homocysteine (umol/L)	7.33	4.44-13.56

**Figure 1:**
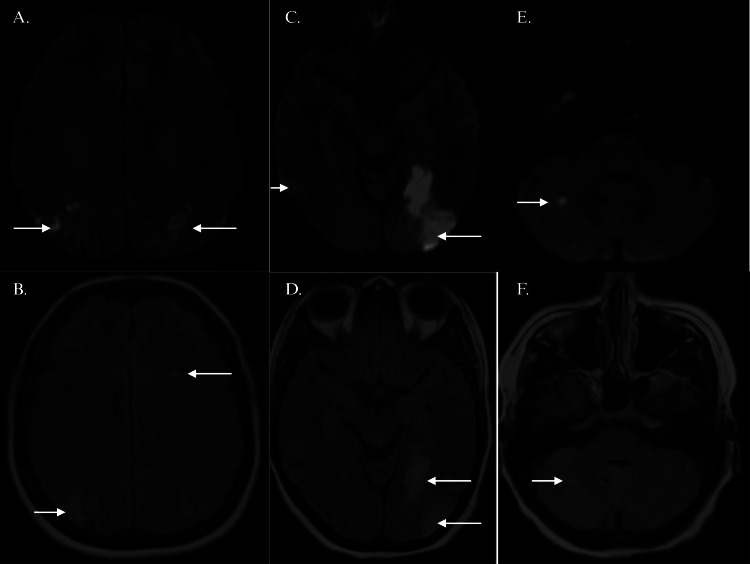
MRI brain showing ischemic infarcts (white arrows) in (A, B) bilateral frontoparietal lobes, (C, D) posterior cerebral artery territory, and (E, F) right cerebellum MRI: magnetic resonance imaging

MRI of the brain showed bilateral posterior cerebral artery (PCA) territory infarcts, along with multiple cardioembolic infarcts in bilateral frontoparietal lobes and right cerebellum, with predominant left-sided involvement (Figures 1A-1F). Transthoracic echocardiogram showed no evidence of lesions or thrombi. However, a transesophageal echocardiogram (TEE) showed a 15 x 4 mm mass in the left atrium, attached to the posterior mitral leaflet. Of note, 24-hour Holter monitoring was not significant.

**Figure 2:**
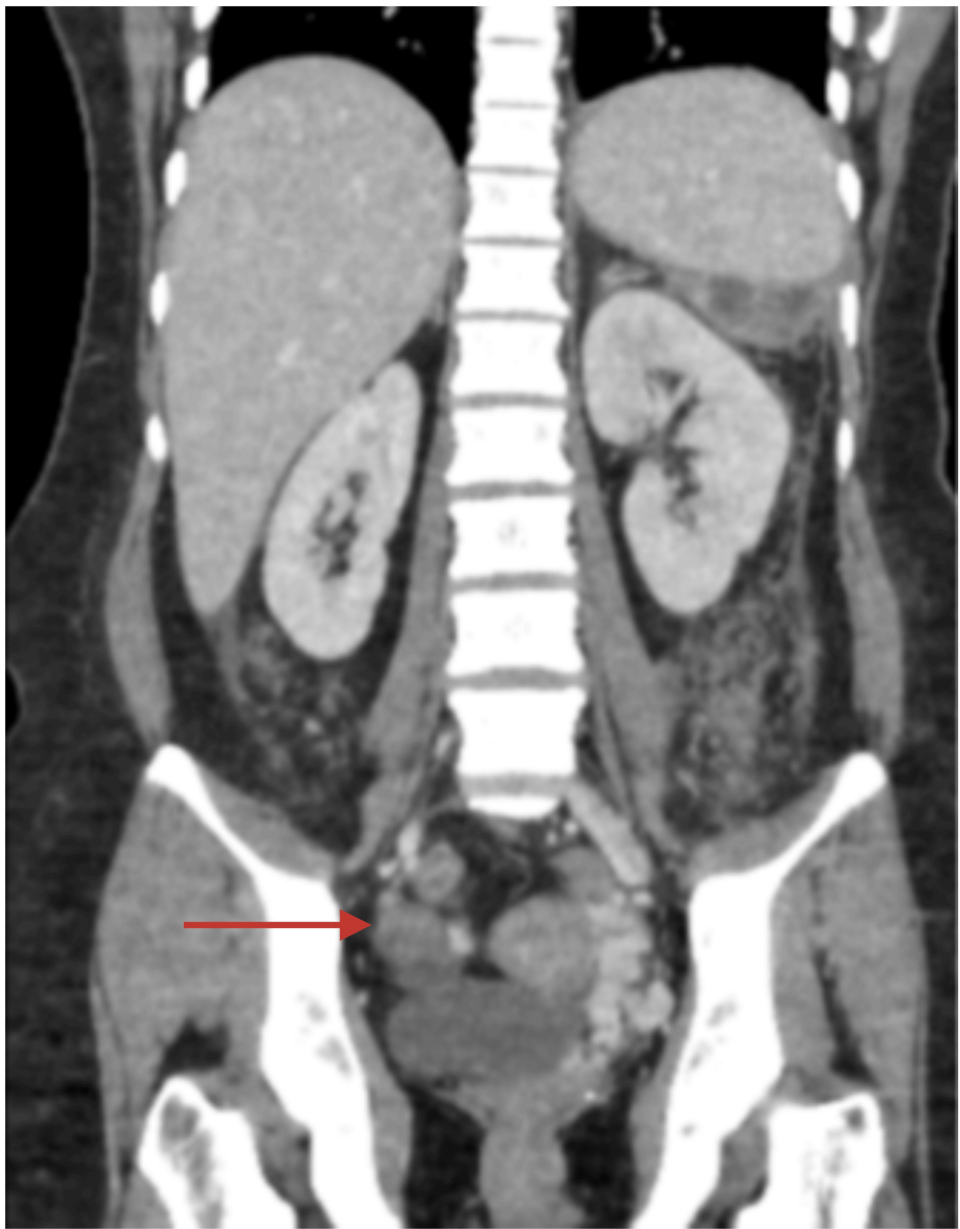
Coronal CT of the abdomen and pelvis showing right ovarian mass (red arrow) CT: computed tomography

Given our likely diagnosis of multiple emboli secondary to a sterile mitral vegetation, we then looked for the cause of her hypercoagulable state. Abdominal CT scan revealed a solitary mass in the right ovary, 15 x 10 x 10 cm in size, with solid and cystic components, suggestive of a neoplasm, with enlarged right iliac lymph nodes (Figure 2). The left ovary was enlarged, measuring 6.2 x 3.3 cm, with endometriotic cysts. Uterine findings were unexceptional apart from a few intramural fibroids and endometrial thickening. Her cancer antigen (CA)-125 levels were elevated significantly at 2000 U/mL (reference range: 0-35 U/mL).

Subsequently, she underwent total abdominal hysterectomy and bilateral salpingo-oophorectomy, with omentectomy and bilateral pelvic and paraaortic node dissection. Histopathological evaluation confirmed grade 2 endometrioid adenocarcinoma of the right ovary. She was deemed to have stage 3 disease and received six cycles of paclitaxel and carboplatin. Her recovery was uncomplicated, except for an occluding right axillary artery thrombus of 1 cm length and another partially occluding left ulnar thrombus, which were managed with appropriate anticoagulation. Her subsequent CA-125 levels were within normal limits. On long-term follow-up, her vision has improved, with no recurrence of the malignancy.

## Discussion

We reported a rare presentation of ovarian endometrioid carcinoma as cardioembolic stroke in a relatively young female patient, highlighting the need to consider occult malignancy as an etiology for stroke in patients without other atherosclerotic risk factors. Cancer-related strokes arise from multiple interplaying mechanisms [1], including hypercoagulability due to tumor-related procoagulants and inflammatory markers [3], cytokine-mediated endothelial damage, and chemotherapy or radiotherapy-induced thrombosis [1,4]. Our patient’s stroke was likely secondary to non-bacterial thrombotic endocarditis (NBTE), stemming from cytokine-mediated endothelial damage.

NBTE is an underdiagnosed condition characterized by sterile valvular vegetations prone to embolization. TEE is the gold standard diagnostic modality. The condition is strongly associated with underlying cancers, particularly adenocarcinomas, and hence its presence merits an occult malignancy workup. In our patient, the presence of a mitral valve mass, with evidence of arterial embolism, without features of infective endocarditis, supported the diagnosis. The treatment involves rapid management of the underlying malignancy and anticoagulation with heparin or low-molecular-weight heparin [5].

Our patient had some subtle clues of occult malignancy. She was iron-deficient without clear contributory reasons, deemed unusual for her socioeconomic and nutritional status. Furthermore, she had elevated inflammatory markers. Prior research has suggested that new-onset anemia may be a marker of occult cancer [6]. Her MRI brain showed the "Three Territory Sign" (TTS), as there was bilateral anterior and posterior territory vascular involvement. TTS is highly specific for cancer-associated stroke, being more frequent in such cases compared to atrial fibrillation-related stroke [2]. 

Overall, stroke as the initial manifestation of malignancy is rare. Previous research has found that only about 0.4% of patients admitted for stroke had underlying malignancy. Of those, approximately one-third were due to NBTE, of which more than half did not have other risk factors for stroke. Among ovarian cancer subtypes, clear cell carcinoma (CCC) is known to be linked to greater thrombotic risk, attributed to tissue factor expression [7]. Reports of stroke due to NBTE associated with ovarian carcinoma all mention CCC as the histologic subtype [8,9]. 

Our patient's case was unique in that she had endometrioid ovarian cancer, a distinct subtype with no prior reports of stroke, to our knowledge. While the prognosis of patients with malignancy-associated stroke is often incredibly poor [1], our patient had a satisfactory outcome. This is likely attributable to early identification of her cancer before distant spread, as well as the relatively favorable histology of endometrioid ovarian cancer [10].

## Conclusions

Stroke may be the first manifestation of ovarian carcinoma, even in histologic subtypes not traditionally linked to hypercoagulability. Malignancy must be considered in the workup of stroke, particularly in young patients without traditional cerebrovascular risk factors, and with the involvement of multiple arterial territories. Clues to malignancy-related stroke include unexplained iron-deficiency anemia, elevated inflammatory markers, and TTS on brain MRI.

## References

[1] Taccone FS, Jeangette SM, Blecic SA (2008). First-ever stroke as initial presentation of systemic cancer. J Stroke Cerebrovasc Dis.

[2] Nouh AM, Staff I, Finelli PF (2019). Three Territory Sign: an MRI marker of malignancy-related ischemic stroke (Trousseau syndrome). Neurol Clin Pract.

[3] De Cicco M (2004). The prothrombotic state in cancer: pathogenic mechanisms. Crit Rev Oncol Hematol.

[4] Kuan AS, Teng CJ, Wu HH (2014). Risk of ischemic stroke in patients with ovarian cancer: a nationwide population-based study. BMC Med.

[5] Itzhaki Ben Zadok O, Spectre G, Leader A (2022). Cancer-associated non-bacterial thrombotic endocarditis. Thromb Res.

[6] Boennelykke A, Jensen H, Østgård LS, Falborg AZ, Hansen AT, Christensen KS, Vedsted P (2022). Cancer risk in persons with new-onset anaemia: a population-based cohort study in Denmark. BMC Cancer.

[7] Uno K, Homma S, Satoh T (2007). Tissue factor expression as a possible determinant of thromboembolism in ovarian cancer. Br J Cancer.

[8] Oueida Z, Scola M (2011). Ovarian clear cell carcinoma presenting as non-bacterial thrombotic endocarditis and systemic embolization. World J Oncol.

[9] Blustein P, Werner S, Sham S, Febles A, Katz H, Villella J (2023). Right middle cerebral artery stroke secondary to ovarian clear cell carcinoma in a 35-year-old: a case report. Case Rep Womens Health.

[10] Chen S, Lu H, Jiang S (2023). An analysis of clinical characteristics and prognosis of endometrioid ovarian cancer based on the SEER database and two centers in China. BMC Cancer.

